# Supplementary material for: Empirical methods for controlling false positives and estimating confidence in ChIP-Seq peaks
Source: BMC Bioinformatics. 2008 Dec 5;9:523. doi: 10.1186/1471-2105-9-523 (PMC2628906; doi:10.1186/1471-2105-9-523)
Supplement: Additional File 1 — A variety of html documents from the USeq web site detailing the available applications, their best usage, output file type descriptions, command line menus, etc. [file 1471-2105-9-523-S1.zip › USeqUserGuides/JavaDoc.zip!/JavaDoc/trans/tpmap/MapSplitter.html]

MapSplitter


|  |  |  |
| --- | --- | --- |
| **Overview** \| **Package** \| **Class** \| **Use** \| **Tree** \| **Deprecated** \| **Index** \| **Help** | |  |
| **PREV CLASS**   **NEXT CLASS** | **FRAMES**    **NO FRAMES**     **All Classes** |  |
| SUMMARY: NESTED \| FIELD \| CONSTR \| METHOD | DETAIL: FIELD \| CONSTR \| METHOD |  |


---


## trans.tpmap Class MapSplitter

```
java.lang.Object
  trans.tpmap.MapSplitter
```

---

``` public class MapSplitter extends java.lang.Object ```

Splits a '.bpmap' text file into different chromosomes, helper script for `TPMapProcessor`.

---

**Constructor Summary**

`MapSplitter(MapFeature[] features,
java.io.File saveDirectory)`
  
          For running in combination with BPMapProcessor


| **Method Summary** | |
| --- | --- |
| `static java.lang.String` | `breakSaveIntensityValues(java.util.ArrayList bpmapInfo, float[][] intensities, java.lang.String fullPathDir)`             Use this method to break a float[][] array into different float[][] by chromosome. |
| `static java.lang.String` | `breakSaveIntensityValues(java.util.ArrayList bpmapInfo, float[] intensities, java.lang.String fullPathBaseName)`             Use this method to break a float[] array into different float[] by chromosome. |
| `static java.lang.String` | `breakSaveIntensityValues(java.util.ArrayList bpmapInfo, int[][] intensities, java.lang.String fullPathDir)`             Use this method to break a int[][] array into different int[][] by chromosome. |
| `static java.lang.String` | `breakSaveIntensityValues(java.util.ArrayList bpmapInfo, int[] intensities, java.lang.String fullPathBaseName)`             Use this method to break a int[] array into different int[] by chromosome. |
| `void` | `breakSavePositions()` |
| `void` | `gatherInfo()` |
| `void` | `splitTPMap()` |

**Methods inherited from class java.lang.Object**

`equals, getClass, hashCode, notify, notifyAll, toString, wait, wait, wait`

**Constructor Detail**

### MapSplitter

```
public MapSplitter(MapFeature[] features,
                   java.io.File saveDirectory)
```

:   For running in combination with BPMapProcessor


**Method Detail**

### splitTPMap

```
public void splitTPMap()
```

---


### breakSavePositions

```
public void breakSavePositions()
```

---


### breakSaveIntensityValues

```
public static java.lang.String breakSaveIntensityValues(java.util.ArrayList bpmapInfo,
                                                        int[] intensities,
                                                        java.lang.String fullPathBaseName)
```

:   Use this method to break a int[] array into different int[] by chromosome.
    Saves the int[] arrays to disk using the fullPathBaseName+ the chromosome name.
    bpmapInfo comes from running the BPMapSplitter on a .bpmap file.

---


### breakSaveIntensityValues

```
public static java.lang.String breakSaveIntensityValues(java.util.ArrayList bpmapInfo,
                                                        float[] intensities,
                                                        java.lang.String fullPathBaseName)
```

:   Use this method to break a float[] array into different float[] by chromosome.
    Saves the float[] arrays to disk using the fullPathBaseName+ the chromosome name.
    bpmapInfo comes from running the BPMapSplitter on a .bpmap file.

---


### breakSaveIntensityValues

```
public static java.lang.String breakSaveIntensityValues(java.util.ArrayList bpmapInfo,
                                                        int[][] intensities,
                                                        java.lang.String fullPathDir)
```

:   Use this method to break a int[][] array into different int[][] by chromosome.
    Saves the int[][] arrays to disk using the fullPathBaseName+ the chromosome name.
    bpmapInfo comes from running the BPMapSplitter on a .bpmap file.

---


### breakSaveIntensityValues

```
public static java.lang.String breakSaveIntensityValues(java.util.ArrayList bpmapInfo,
                                                        float[][] intensities,
                                                        java.lang.String fullPathDir)
```

:   Use this method to break a float[][] array into different float[][] by chromosome.
    Saves the float[][] arrays to disk using the fullPathBaseName+ the chromosome name.
    bpmapInfo comes from running the TPMapSplitter on a .tpmap file.

---


### gatherInfo

```
public void gatherInfo()
```


---


|  |  |  |
| --- | --- | --- |
| **Overview** \| **Package** \| **Class** \| **Use** \| **Tree** \| **Deprecated** \| **Index** \| **Help** | |  |
| **PREV CLASS**   **NEXT CLASS** | **FRAMES**    **NO FRAMES**     **All Classes** |  |
| SUMMARY: NESTED \| FIELD \| CONSTR \| METHOD | DETAIL: FIELD \| CONSTR \| METHOD |  |


---
